# Isolation and characterization of extracellular vesicles from human milk for potential use as a dietary supplement in clinical research with preterm infants

**DOI:** 10.3389/fnut.2026.1764928

**Published:** 2026-03-20

**Authors:** Jose Luis Moreno-Casillas, Laura Ripoll-Seguer, Emmanuel Rumba-Matano, Anna Parra-Llorca, María Cernada, María Jesús Vaya, Eduardo López Briz, Ana María Padilla-López, Ana Gil Brusola, Bernhard Lendl, Guillermo Quintás, María Gormaz, Julia Kuligowski

**Affiliations:** 1 Neonatal Research Group, Health Research Institute La Fe (IIS La Fe), València, Spain; 2 Servicio de Análisis de Vesículas Extracelulares (SAVE), Health Research Institute La Fe (IIS La Fe), València, Spain; 3 Spanish Network in Maternal, Neonatal, Child and Developmental Health Research (RICORS- SAMID) (RD24/0013/0014), Neonatal Research Group, Health Research Institute La Fe, València, Spain; 4 Division of Neonatology, University and Polytechnic Hospital La Fe (HULAFE), València, Spain; 5 Blood Transfusion Center from the Valencian Community, València, Spain; 6 Department of Hospital Pharmacy, University and Polytechnic Hospital La Fe, València, Spain; 7 Department of Microbiology, University and Polytechnic Hospital La Fe, València, Spain; 8 Institute of Chemical Technologies and Analytics, Technische Universität Wien, Vienna, Austria; 9 Health and Biomedicine, Leitat Technological Center, Terrassa, Spain

**Keywords:** donor human milk (DHM) supplementation, human milk-derived extracellular vesicles (HMEVs), necrotizing enterocolitis (NEC), neonatal intestinal protection, preterm infant nutrition

## Abstract

**Background/objectives:**

Human milk (HM) is the gold standard for neonatal nutrition, providing essential macronutrients and bioactive compounds that promote immune and gastrointestinal development. Among these components, HM-derived extracellular vesicles (HMEVs) are emerging as key mediators of intestinal maturation and protection against necrotizing enterocolitis (NEC). HMEVs carry miRNAs, proteins, and bioactive lipids that resist digestion and modulate critical signaling pathways in the immature gut, making them particularly relevant for preterm infants.

**Methods:**

This study describes the development and adaptation of a robust, scalable workflow for isolating HMEVs from donor HM for potential use as a nutritional supplement. An initial laboratory-scale isolation protocol was successfully scaled up to a sterile, clinically compatible process. Key modifications included increasing ultracentrifugation speed, eliminating filtration, and replacing phosphate-buffered saline (PBS) with a resuspension medium suitable for nutritional applications.

**Results:**

The scaled procedure increased the processed milk volume from 25 mL to 63 mL while reducing the HMEVs isolation time from 14 hs to 7.7 h. Tunable Resistive Pulse Sensing (TRPS) and ExoView^Ⓡ^ analysis showed that these modifications led to an approximate 2-fold increase in EV recovery following centrifugation speed optimization. In addition, omission of filtration yielded a 4.3-fold increase in HMEV recovery. The isolated HMEVs exhibited high purity, reaching up to 3.98 × 10^13^ particles/g protein. The final product was resuspended in 5% glucose solution, chosen for its physiological compatibility, favorable osmolarity (< 380 mOsmol/kg), and ability to preserve EV stability for up to 30 days at −80 °C. The HMEV preparation was produced under sterile conditions, and endotoxin testing returned negative results.

**Conclusions:**

This optimized and scalable method enables the safe and efficient isolation of HMEVs for use in neonatal nutritional supplements. These findings establish methodological groundwork for future translational studies of milk-derived EVs aimed at supporting intestinal development and immune protection, and potentially reducing the risk of NEC in preterm infants.

## Introduction

Preterm infants, particularly those with very low birth weight (< 1,500 g), are at high risk of severe morbidities due to the immaturity of their immune and gastrointestinal systems (1). Among these, necrotizing enterocolitis (NEC) remains a major cause of neonatal mortality and long-term sequelae. NEC primarily affects infants born before 32 weeks of gestation and is characterized by intestinal barrier disruption, uncontrolled inflammation, and, in advanced stages, extensive tissue necrosis. Despite advances in neonatal intensive care, NEC continues to present a significant clinical challenge (1, 2). Given the early onset and rapid progression of NEC, the development of preventive strategies may offer greater clinical benefit for this highly vulnerable population (1, 3).

Human milk (HM) is a key protective factor against NEC, largely attributed to its unique composition of nutrients, immunomodulatory components, and bioactive molecules that promote intestinal maturation and immune development (4–8). When mother's own milk (MOM) is unavailable, donor human milk (DHM) is the recommended alternative; however, its bioactive profile is reduced by classical Holder pasteurization, which has been shown to cause a marked loss of bioactive components (9, 10).

Among the emerging bioactive components of HM, HM-derived extracellular vesicles (HMEVs) have gained increasing attention due to their ability to modulate immune responses and maintain intestinal homeostasis. These nanoscale particles, primarily derived from mammary epithelial cells, carry a complex molecular cargo, including microRNAs, lipids, and functional proteins, and are known to resist digestive degradation and interact with epithelial and immune cells in the neonatal gut (11–15). Previous studies have shown that HMEVs reduce intestinal inflammation, preserve epithelial barrier function, and improve outcomes in murine models of NEC (13, 16–19), supporting their therapeutic potential to improve intestinal health. However, the use of HMEVs as dietary supplements in preterm infants remains largely unexplored (20, 21).

This study describes the development of a workflow for the isolation of HMEVs from DHM, intended for future clinical use as a nutritional supplement to be investigated for its potential role in reducing the risk of NEC in preterm neonates. The workflow employs a high-capacity Beckman 50.2 Ti rotor (63 mL) for HMEV isolation, within a process designed to meet clinical requirements for food safety. Using a multi-step ultracentrifugation (UC)-based protocol (22) as reference, quality control measures included the analysis of isolated HMEVs concentration and purity, molecular profiling, and tetraspanin surface co-localization analysis. Furthermore, the solvent compatibility for enteral administration, osmolarity, total protein content, microbiological safety, and endotoxicity of the HMEV preparations were rigorously assessed.

## Materials and methods

### Reagents and materials

Ultrapure water was obtained using a Milli-Q integral water purification system (Merck Millipore, Darmstadt, Germany). Phosphate-buffered saline (PBS) tablets (pH 7.2–7.6, 1 tablet/200 mL) were acquired from Sigma-Aldrich Química SL (Madrid, Spain) and filtered using 47 mm nylon membrane filters (0.22 μm pore size; Scharlau, Barcelona, Spain) with a vacuum filtration system. Clinically approved infusion-grade solutions, including 0.9% (w/v) saline (Fresenius Kabi, Barcelona, Spain) and 5% (w/v) glucose (Laboratorios Grifols, Barcelona, Spain), were supplied by B. Braun Medical S.A. (Barcelona, Spain). Leprechaun™ Exosome Human Tetraspanin kits (Ref. NAV251-1044) with immobilized tetraspanin antibodies (i.e., CD63, CD81, and CD9) were provided by Izasa Scientific (Barcelona, Spain). NP250 nanopores and CPC200 carboxylate polystyrene calibration particles (200 nm, 8.2 × 10^11^ particles/mL) for Tuneable Resistive Pulse Sensing (TRPS) measurements were acquired from Izon Science Europe Ltd (Lyon, France). Bovine serum albumin (BSA) (0–6 g/L) for protein calibration were obtained from Sigma-Aldrich Química SL (Madrid, Spain). The bicinchoninic acid (BCA) protein assay kit was purchased from Thermo Scientific (Waltham, MA, USA). Seventy percent ethanol (VWR Chemicals, Radnor, PA, USA) was used for surface and hand disinfection. Surfa Safe (Laboratoires ANIOS, Lezennes, France), a hospital-grade surfactant-based cleaner, was employed for routine decontamination of the working environment prior to sterile procedures.

### HM collection

The study was approved by the Ethics Committee for Biomedical Research at the Health Research Institute La Fe (Valencia, Spain; 2023-1191-1) and conducted in accordance with the Declaration of Helsinki. All HM donors signed an informed consent form prior to their participation in the study. Two sources of DHM were used: (i) raw milk for laboratory-scale experiments and scalability testing obtained from the Human Milk Bank at the University and Polytechnic Hospital La Fe (Valencia, Spain) and (ii) pasteurized donor milk for microbiological safety testing provided by the Human Milk Bank of the Transfusion Center of the Valencian Community (Spain). Milk samples were collected following complete breast expression, according to the hygiene protocols approved at the milk bank: (i) thorough hand washing with soap and water, followed by drying and hand disinfection; (ii) cleansing of the breast area with water and sterile gauze, without the use of creams or lotions; (iii) milk extraction using sterilized pumps into sterile containers.

### Optimization of HMEV isolation

Five UC protocols (CTRL and methods 1–4) summarized in Table 1 were tested using raw HM pooled from multiple donors. All experiments were performed on a unique pooled HM sample to facilitate the comparison across protocols. Experiments were conducted with a Beckman Coulter Optima XE UC and a Type 50.2 Ti fixed-angle rotor using 26 mL polycarbonate tubes with aluminum caps (Beckman Coulter, USA). In all methods, the first step consisted of an initial centrifugation at 3.000 × g for 10 min to remove fat and cellular debris.

**Table 1:** Overview of the control (CTRL) and processing methods (1–4) for the isolation of HMEVs from DHM, involving different centrifugation steps for fat removal and debris clearance.

**Isolation time and steps**	**CTRL**	**Method 1**	**Method 2**	**Method 3**	**Method 4**
Defatting and cell removal	3000 x *g* 10 min	3000 x *g* 10 min	3000 x *g* 10 min	3000 x *g* 10 min	3000 x *g* 10 min
Protein and cell debris removal	12.000 x *g* 60 min	12.000 x *g* 60 min	12.000 x *g* 60 min	12.000 x *g* 60 min	12.000 x *g* 60 min
	12.000 x *g* 60 min	–	12.000 x *g* 60 min	12.000 x *g* 60 min	–
HMEV isolation	105.000 x *g* 120 min	105.000 x *g* 120 min	105.000 x *g* 120 min	105.000 x *g* 120 min	105.000 x *g* 120 min
	105.000 x *g* 120 min	105.000 x *g* 120 min	105.000 x *g* 120 min	–	–
	105.000 x *g* 120 min	105.000 x *g* 120 min	**–**	**–**	**–**
Isolation time (h)	14	11.9	10.9	7.7	6.4

^*^Processing time required for the processing of three samples handled in parallel by two operators and using a rotor capacity of six tubes for UC. All conditions were tested employing 25 mL of HM and a final resuspension volume of 200 μL.

Then, as summarized in Table 1, the CTRL reference protocol included two centrifugations at 12.000 × g for 60 min each, followed by 0.45 μm syringe filtration and three UC cycles at 105.000 × g for 120 min, leading to a total processing time of 14 h. Methods 1–4 were designed to assess the impact on HMEVs yield, purity, and sample throughput of modifying either the protein and cell debris removal step, the EV isolation step, or both.

As detailed in “Characterization and quality control of isolated HMEVs,” several quality control protocols were implemented to evaluate the extent to which the removal of ultracentrifugation steps affects the purity of isolated HMEVs, including protein co-localization analysis, particle size and concentration measurements, molecular fingerprinting, and total protein quantification.

### Scaling-up and workflow adaptations to support future translational studies

HMEV isolation method 3 was used for the up scaling of the HMEV isolation procedure. Accordingly, pooled raw DHM was ultracentrifuged using a Beckman Coulter Optima XE UC equipped with a Type 50.2 Ti rotor for lab-scale isolation (26.3 mL tubes, Beckman Coulter, USA) and 45 Ti rotor for milk-bank isolation (94 mL polypropylene tubes with aluminum caps, Beckman Coulter, USA). For working at milk-bank scale, to preserve pelleting efficiency while maintaining the same centrifugation time, two rotor speeds were tested: (i) maintaining the *g*-force with respect to the laboratory method at 105.000 × *g* and (ii) increasing the *g*-force to 160,000 × *g* in accordance with Beckman Coulter guidelines (23). Final EV pellets were resuspended in 500 μL of 5% glucose solution.

Filtration was re-evaluated due to practical limitations encountered during up-scaling of the protocol. Laboratory protocols involve filtration of the entire milk pool using 0.45 μm nylon syringe filters (Labbox, Spain). For milk-bank scale, 0.45 μm capsule filters (Membrane Solutions, USA) coupled to a peristaltic pump (Labbox, Spain) were tested to enable continuous operation and reduce clogging.

Finally, to examine the tolerance and safety of the nutritional preparation, HMEVs isolated by method 3 from DHM samples obtained from the milk bank were subjected to additional quality control tests, including osmolarity, endotoxin levels, microbiological safety, and total protein content assessments. All procedures are detailed in “Characterization and quality control of isolated HMEVs.” Strict aseptic practices were followed, including thorough handwashing, application of 70% ethanol to hands and forearms, donning of sterile disposable surgical gowns and gloves, and a two-step disinfection of the laminar flow hood with surfactant-based cleaners and with 70% ethanol. Furthermore, the laminar flow hood was exposed to ultraviolet irradiation for 15 min prior to use to minimize potential contamination. Special care was taken to maintain sterility during the introduction of samples, reagents, and materials. All new items brought into the sterile field were disinfected with ethanol, including gloves when replacement. Microbiological cultures were performed both before and after sample handling to monitor potential microbiological contamination. The increase in total protein content was quantified in triplicate by comparing an HM sample before and after supplementation with HMEVs at a ratio of 1:125 (HMEVs:HM), reflecting the supplementation conditions intended for use in future clinical studies.

### Characterization and quality control of isolated HMEVs

#### Protein co-localization analysis

Tetraspanin profiling was performed using the ExoView^®^ R200+ system from NanoView Biosciences, Inc. (Brighton, MA, USA) with Leprechaun chips functionalized with CD9, CD63, and CD81 from Unchained Labs (Pleasanton, CA, USA). HMEV samples were diluted 1:100 or 1:1,000 in PBS and incubated overnight at room temperature with 50 μL of diluted sample. After incubation, fluorescent detection antibodies were applied following the manufacturer's guidelines. Data acquisition and analysis were carried out using the ExoView Analyzer software (NanoView Biosciences).

#### Particle size and concentration

HMEV size and concentration were measured using the Exoid TRPS platform (Izon Science Ltd., Christchurch, New Zealand). A single NP250 nanopore was used at 1,000 and 800 Pa, with a nanopore stretch between 46–48 mm. CPC200 particles and HMEV samples were diluted (i.e., 1:1,000 and 1:2,000, respectively) in the same filtered PBS. At least 500 particle events were recorded per run under identical settings. Data were acquired and analyzed using Izon Control Suite software (v1.0.2.32).

#### Molecular fingerprinting

Attenuated Total Reflectance-Fourier Transform Infrared (ATR-FTIR) spectroscopy measurements were performed on an Alpha II spectrometer from Bruker Optics GmbH (Ettlingen, Germany) equipped with a DTGS detector, CenterGlow™ IR source, and a single-reflection diamond ATR crystal. A 2 μL aliquot of each HMEV suspension was deposited onto the crystal surface and air-dried for 5 min at room temperature under mild airflow. Spectra were recorded in the 4,000–400 cm^−1^ range with a resolution of 4 cm^−1^, averaging 32 scans per sample. A PBS spectrum collected under identical conditions was subtracted as background, and the ATR interface was thoroughly cleaned with Milli-Q water between measurements to avoid sample carryover.

ATR-FTIR profiling provided detailed molecular fingerprints of the samples, allowing comparative evaluation of protein, lipid, and nucleic acid content across different isolation methods. For β-sheet-to-α-helix ratio (β/α) calculations, the second derivative of the amide I band was computed to enhance spectral resolution, and peak areas were integrated at characteristic wavenumber ranges: 1,653 cm^−1^ for α-helix, and 1,633 cm^−1^ and 1,644 cm^−1^ for β-sheet structures. The β/α was calculated as the combined area under the β-sheet peaks divided by the α-helix peak area. The protein-to-lipid ratio (PLR) was derived from the raw ATR-FTIR spectrum by integrating the area of the amide I band (1,724–1,591 cm^−1^) as a surrogate for protein content and the area of the CH-stretching region (2,763–2,985 cm^−1^) as a surrogate for lipid content. These spectral ratios served as indirect indicators of sample complexity and aided assessing potential co-isolation of non-vesicular components.

#### Total protein quantification

Protein content was determined by BCA assay, performed following the manufacturer's instructions, which, despite potential interference from some buffer components, provides a widely used and consistent measure for comparative purposes. Samples were incubated with BCA working reagent at 37°C for 30 min, and absorbance was measured at 562 nm using a microplate reader. BSA was used as the standard for calibration. For purity analysis, the particle-to-protein ratio was calculated. For this, particle concentration obtained by TRPS measurements was divided by the total protein concentration determined by the BCA assay, providing an estimate of vesicle purity expressed as number of particles per gram of protein.

#### Osmolarity testing

Osmolarity was measured using a Fiske Micro-Osmometer (Advanced Instruments, USA), based on freezing point depression. The device operated within a 0–3,000 mOsm/kg range with ±2 mOsm/kg precision. Each measurement was performed with 20 μL of sample, including both the HMEV extract and the defatted HM (obtained by centrifugation at 3.000 × g for 10 min) used as a control. Calibration was carried out using certified standards (290 and 1,000 mOsm/kg), and a calibration curve was performed weekly. A 500 mOsm/kg control standard was used for quality control. Storage was at 2–8 °C and ambient temperature during analysis was controlled (21–23 °C).

#### Microbiological testing

Microbiological testing was performed to verify the absence of a significant bacterial load of pasteurized DHM and the final EV isolate. Prior to processing, each batch of DHM underwent routine microbiological screening. Analyses were carried out by the Microbiology Unit at the University and Polytechnic Hospital La Fe (Valencia, Spain), following validated clinical protocols and under aseptic conditions in a certified biosafety cabinet. Briefly, the EV isolate was inoculated in a liquid enrichment medium (thioglycolate) and incubated for 24–48 h at 35–37°C under 5–7% CO_2_. After incubation, a blind passage was performed onto aerobic solid media (AS) to assess bacterial growth. Batches yielding are considered suitable for consumption, as their spores are thermoresistant.

#### Endotoxicity assay

Endotoxin levels were measured using a chromogenic LAL assay. Samples, standards, and blanks (50 μL each, in triplicate) were added to a 96-well plate pre-incubated at 37 ± 1°C. After addition of 50 μL of Amebocyte Lysate Reagent, the plate was incubated (T1), followed by 100 μL of chromogenic substrate and a second incubation (6 min). The reaction was stopped with 25% acetic acid, and absorbance was read at 405 nm. Endotoxin concentrations were calculated from a standard curve (R^2^ ≥ 0.98) using blank-corrected values.

#### Transmission electron microscopy (TEM)

Samples of isolated vesicles were prepared for TEM visualization. An aliquot of 10 μl of the vesicle suspension, resuspended in 0.1 M PBS, was deposited onto a piece of Parafilm. Carbon-coated copper grids were then placed on these suspension drops and incubated for 10 min to allow for vesicle adhesion. The grids with the adherent vesicles were washed by immersion in a drop of 0.1 M PBS, and subsequent additional fixation was performed using 1% glutaraldehyde for 5 min. Following fixation, the grids were washed thoroughly with distilled water and subjected to contrast staining with 2% uranyl acetate before being embedded in methylcellulose. Excess fluid was removed, and the grids were allowed to dry completely prior to observation. Samples were examined using an FEI Tecnai G2 Spirit Transmission Electron Microscope (ThermoFisher Scientific company, Oregon, USA). Micrographs were captured with a Xarosa digital camera (EMSIS GmbH, Münster, Germany), controlled by the Radius software (Version 2.1).

## Results

### Selection of the HMEV isolation workflow

**Figure 1:**
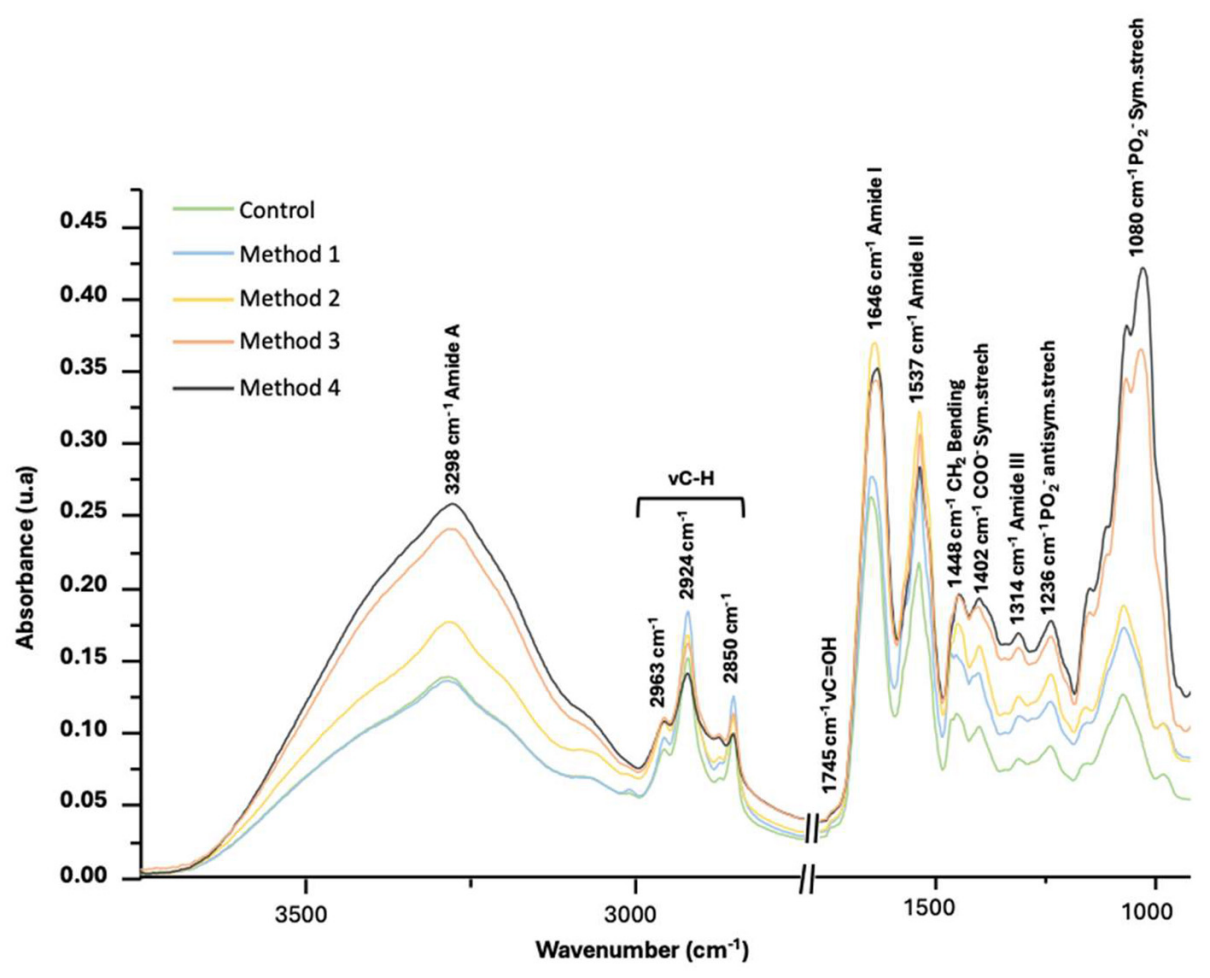
ATR-FTIR spectra of HMEVs isolated by different UC methods. All HMEV isolates were obtained from an initial volume of 25 mL of HM and resuspended in a final volume of 200 μL of PBS.

To characterize the composition and structural features of EV isolates, ATR-FTIR spectroscopy was combined with quantitative purity metrics. Spectral analysis revealed increased absorbance in methods 3 and 4 of characteristic bands such as 1,236 cm^−1^ (phospholipids/nucleic acids), 1,314 cm^−1^ (proteins), 1,402 cm^−1^ (fatty acids/amino acids), 1,448 cm^−1^ (lipid acyl chains), and across the CH-stretching region (2,800–3,000 cm^−1^), consistent with greater molecular complexity (see Figure 1).

To support this observation, two complementary FTIR-derived indicators were evaluated (i.e., the PLR and the β/α), with results shown in Table 2. The PLR has been proposed as a useful spectroscopic proxy for EV purity, with elevated values typically reflecting the co-isolation of protein-rich, non-vesicular contaminants, such as soluble proteins or protein aggregates (24). Similarly, increased β/α ratios may result from unordered or aggregated proteins that are not part of the vesicle structure but are retained due to insufficient purification (25). Both metrics were significantly higher in methods 2, 3, and 4 compared to the control, indicating varying degrees of co-isolated material.

**Table 2:** Summary of measured quality control spectroscopic parameters (mean ± SD).

**Metric**	**Control**	**Method 1**	**Method 2**	**Method 3**	**Method 4**
β/α	1.62 ± 0.02	1.62 ± 0.13	2.35 ± 0.07^*^	2.17 ± 0.03^*^	1.95 ± 0.17^*^
PLR	1.34 ± 0.05	1.28 ± 0.02	1.63 ± 0.02^*^	1.51 ± 0.04^*^	1.44 ± 0.02^*^

^*^Statistically significant differences compared to control (Tukey test, *p*-value < 0.05).

**Figure 2:**
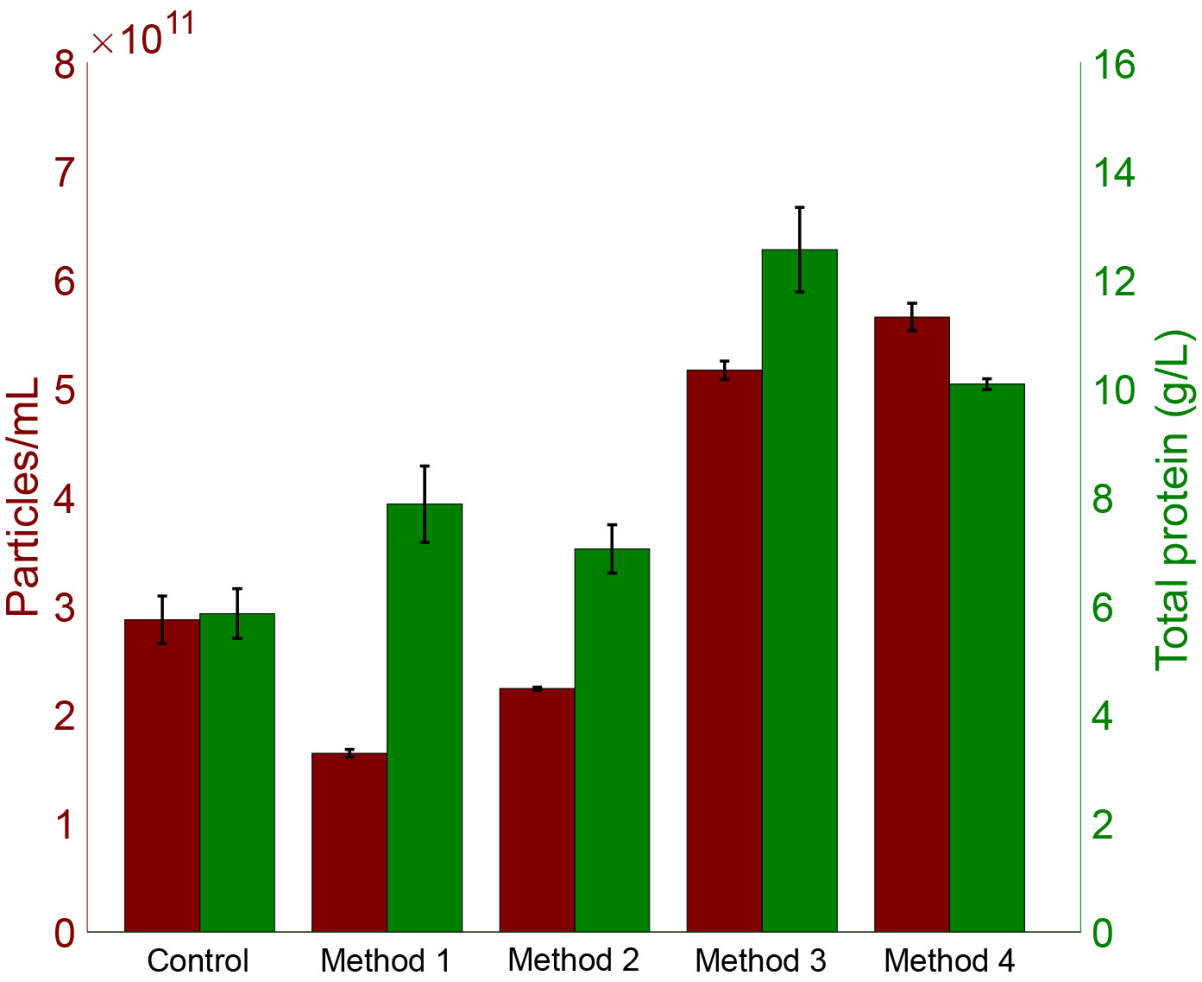
Comparison of particle concentration (particles/mL, TRPS) and total protein content (g/L, BCA assay) across different HMEV isolation methods.

To complement the spectroscopic assessment with a more quantitative perspective, particle-to-protein ratios were calculated (26) with the corresponding protein and particle values shown in Figure 2 (see size distribution histograms in Supplementary Figure S1). Method 4 yielded the highest value (6.52 × 10^13^ ± 1.32 × 10^13^ particles/g protein), followed by method 3 (4.13 × 10^13^ ± 2.93 × 10^12^) and the control (4.91 × 10^13^ ± 1.71 × 10^12^). Methods 2 and 1 showed lower ratios, with method 1 being significantly different from the control (*p*-value = 0.03, Tukey test). Elevated protein levels likely contributed to the reduced purity observed in spectroscopic profiles, and higher particle counts detected by TRPS in methods 3 and 4 indicated improved vesicle recovery in these simplified protocols. Method 3 retained two 12,000 × g centrifugation steps, which were omitted in method 4. Processing time was 7.7 h compared to 14 h in the control.

**Figure 3:**
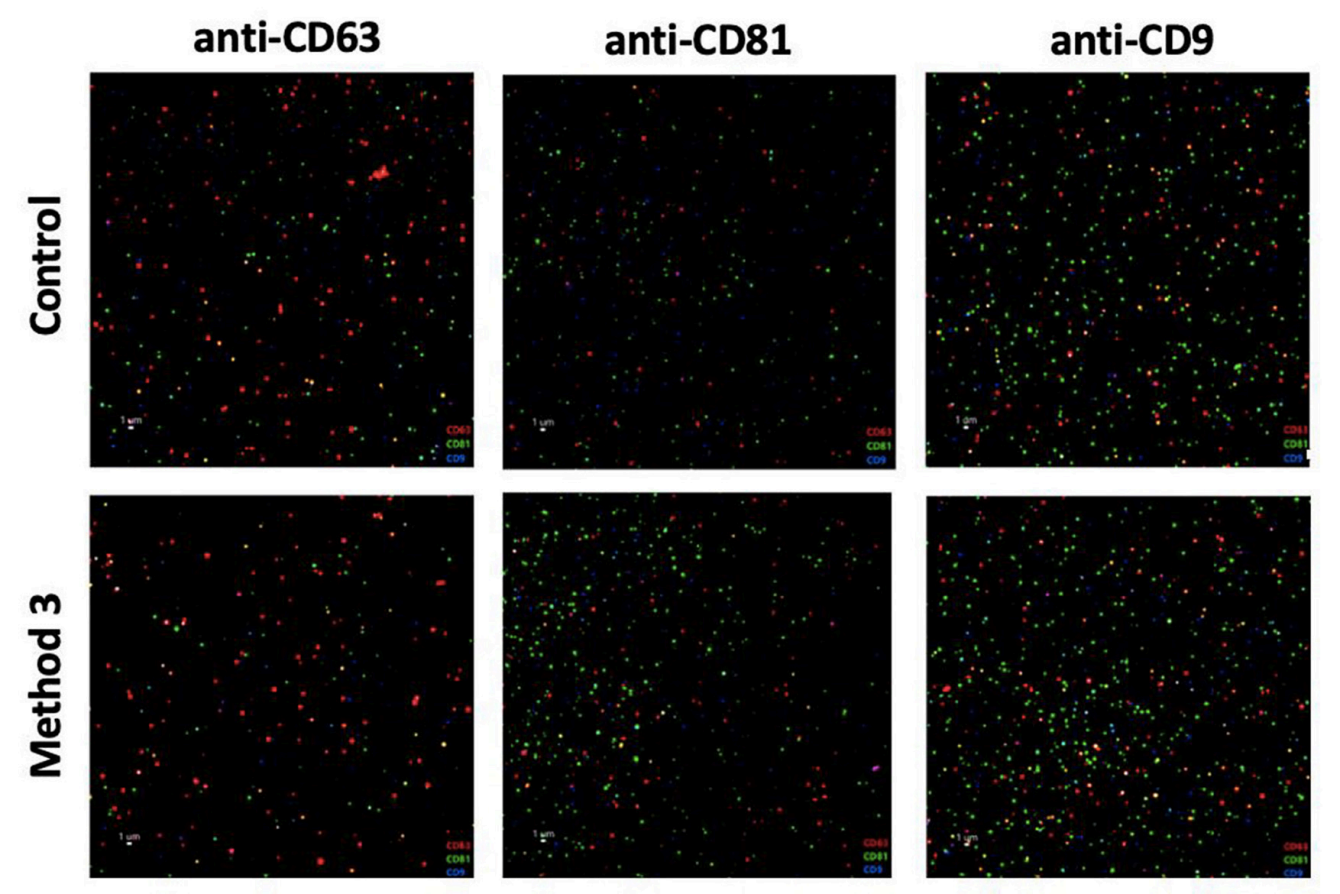
Fluorescence microscopy images of HMEVs immunolabeled with anti-CD63 (red), anti-CD9 (blue), and anti-CD81 (green) antibodies.

To further validate its performance and confirm the comparability of the isolated vesicle population with that of the control method, a DHM sample was isolated and characterized using ExoView^®^. This immunoassay-based platform enables the selective detection and phenotyping of individual EVs based on tetraspanin expression, offering higher specificity and resolution than bulk quantification techniques. ExoView^®^ analysis confirmed the structural integrity and identity of HMEVs isolated using method 3. Co-localization of CD9, CD81, and CD63 surface markers (see Figure 3) revealed vesicle profiles closely aligned with those from the reference method. Furthermore, the surface marker composition of HMEVs captured by CD63, CD81, and CD9 antibodies was analyzed to compare the relative abundance of each tetraspanin. As shown in Supplementary Figure S2, method 3 (red) and the control method (blue) exhibited highly similar profiles. A slightly higher proportion of CD81^+^ EVs was observed in the control, whereas method 3 showed a modest increase in CD63^+^ vesicles when using CD63 for EV capturing. Furthermore, HMEV presence was confirmed by TEM (Supplementary Figure S3), showing vesicles with typical morphology surrounded by casein micelles.

**Figure 4:**
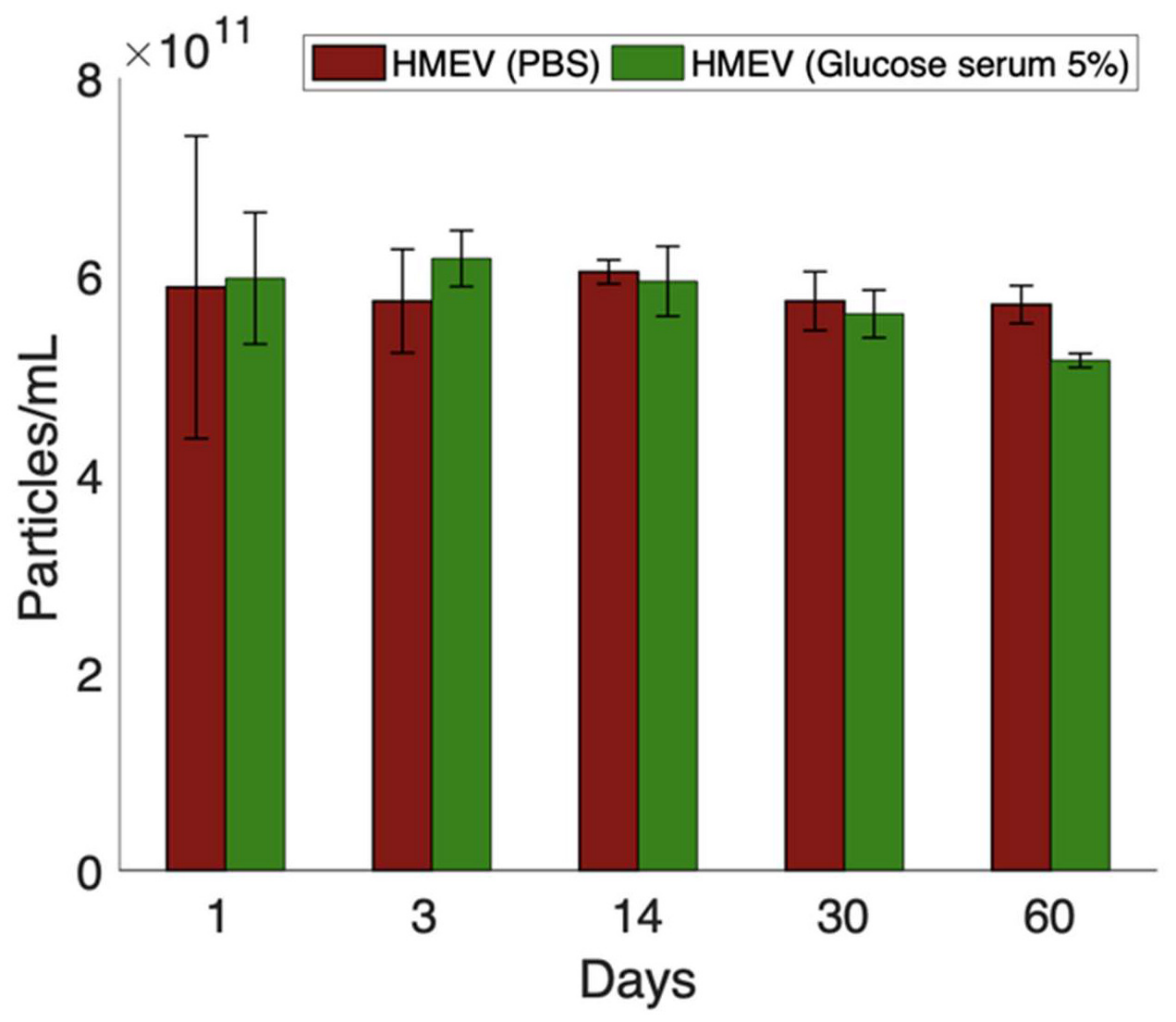
Stability of HMEVs resuspended in PBS and 5% glucose solution over 60 days. Particle concentration was measured at indicated time points by TRPS. Data shows mean ± SD from three replicates. No significant differences were observed between days (ANOVA, *p*-value > 0.05), indicating stable particle concentration under the tested storage conditions.

The stability of HMEVs resuspended in two solutions suitable for enteral administration, i.e., saline (0.9% NaCl) and glucose solution (5%), was initially considered. However, saline was excluded from the stability study due to poor and inconsistent resuspension of HMEV pellets, which resulted in unreliable particle recovery. Stability was then evaluated over a 60-day period in 5% glucose solution, using PBS as a reference. Figure 4 depicts retrieved particle concentrations over time. As expected, no change in the particle concentrations was observed in PBS throughout the 60-day period. Particle concentration values in 5% glucose solution solutions showed a slightly decreasing trend after 14 days; however, the ANOVA test showed no statistically significant differences between days, indicating preserved particle stability without detectable aggregation or loss under these storage conditions.

### Up-scaling from laboratory use to nutritional supplements

To assess translational feasibility, it was necessary to scale up the HMEV isolation process from a laboratory scale to a larger milk bank-compatible scale. For this purpose, UC was evaluated using two different rotor configurations: the 50.2 Ti rotor (25 mL tubes, lab-scale) and the 45 Ti rotor (63 mL tubes, milk bank-scale). Initially, using the same DHM pool and g-force with both rotors to ensure comparability, the mean particle concentrations achieved using the lab-scale 50.2 Ti rotor were (3.1 ± 1.0) × 10^11^ HMEVs/mL, corresponding to (1.3 ± 0.4) × 10^1^ HMEVs/mL per initial volume of HM and (2.4 ± 0.8) × 10^11^ HMEVs/mL in the extract corresponding to (3.8 ± 1.2) × 10^8^ HMEVs/mL per HM when scaling up to the larger 45 Ti rotor. Scaling up to larger volumes led to an approximate 70% reduction in EV recovery. When increasing the centrifugation speed to 160,000 × g, vesicle recovery improved by approximately 1.7-fold compared to the reference condition (Table 3).

**Table 3:** Summary of rotor type, filtration status, centrifugation speed, and HMEV concentrations obtained under different large-scale isolation conditions.

**Condition**	**Filtered**	**Speed x *g***	**(HMEVs/mL) × 10^12^ extract**	**(Condition/reference) ratio**
1	Yes	105.000	1.05	1
2	Yes	160.000	1.77	1.69
3	No	105.000	4.5	4.29

Secondly, the filtration step was reconsidered. In previous experiments, the HM samples had been filtered using 0.45 μm syringe filters under the same conditions as the laboratory process. However, filtering 63 mL by syringe introduced significant sample handling and time investment. Alternatively, capsule filters with 0.45 μm pore size, operated via a peristaltic pump, were tested. These filters allowed continuous filtration, reducing both sample handling time and the risk of filter clogging. Nonetheless, capsule reuse after cleaning with hot water and re-sterilization led to residual organic material buildup (likely lipids) between uses, imposing a potential risk of contamination of subsequent batches. Given that filtration primarily serves to enhance purity for analytical purposes, it was decided to omit this step to further simplify the protocol. As shown in Table 3, eliminating the filtration step led to a 4.3-fold increase in vesicle concentration.

**Figure 5:**
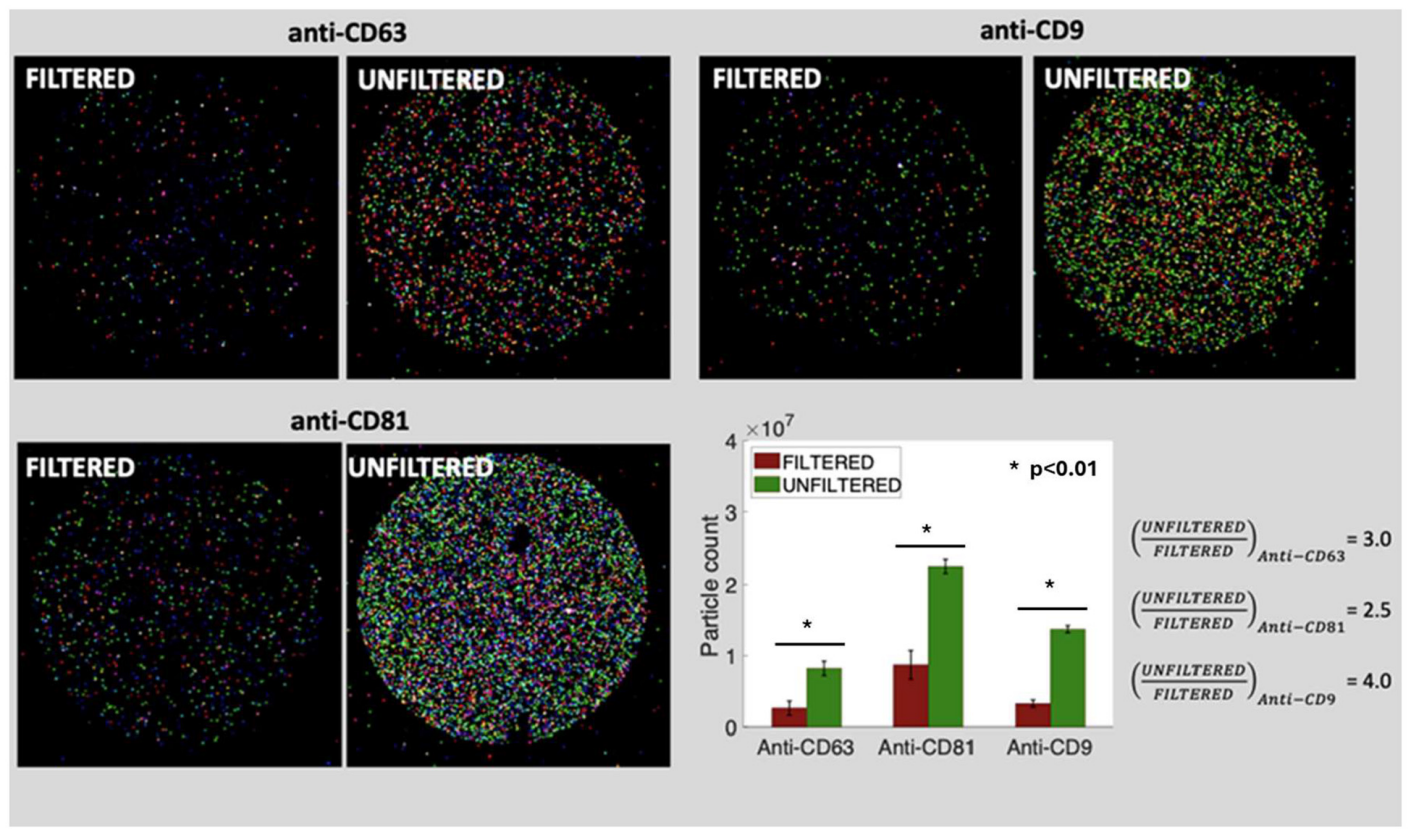
Co-localization analysis of HMEVs in filtered and unfiltered HMEV isolates. Fluorescence microscopy images of HMEVs immunolabeled with anti-CD63 (red), anti-CD9 (blue), and anti-CD81 (green) antibodies in unfiltered and filtered samples captured with anti-CD63 **(top, left)**, anti-CD9 (top, right), and anti-CD81 (bottom, left) antibodies and quantification of particle counts shows a significant decrease in EV recovery upon filtration for all three markers (Wilcoxon rank-sum test, *p*-value < 0.01) **(bottom, right)**. ^*^indicate values with *p* < 0.05, representing statistically significant differences.

Given the marked rise in particle concentration detected by TRPS, ExoView^®^ analysis was conducted to determine whether this increase reflected true vesicle recovery or the co-isolation of larger milk-derived components. Comparing filtered and non-filtered samples revealed a 3-fold increase in vesicle counts under non-filtered conditions (Figure 5). Moreover, colocalization profiles of tetraspanins for the immunocaptures with CD63, CD81, and CD9 were evaluated (Supplementary Figure S4). An overview comparing both the laboratory scale and the milk bank scale procedures is provided in Supplementary Figure S5.

### Quality-control readouts for the optimized workflow

**Figure 6:**
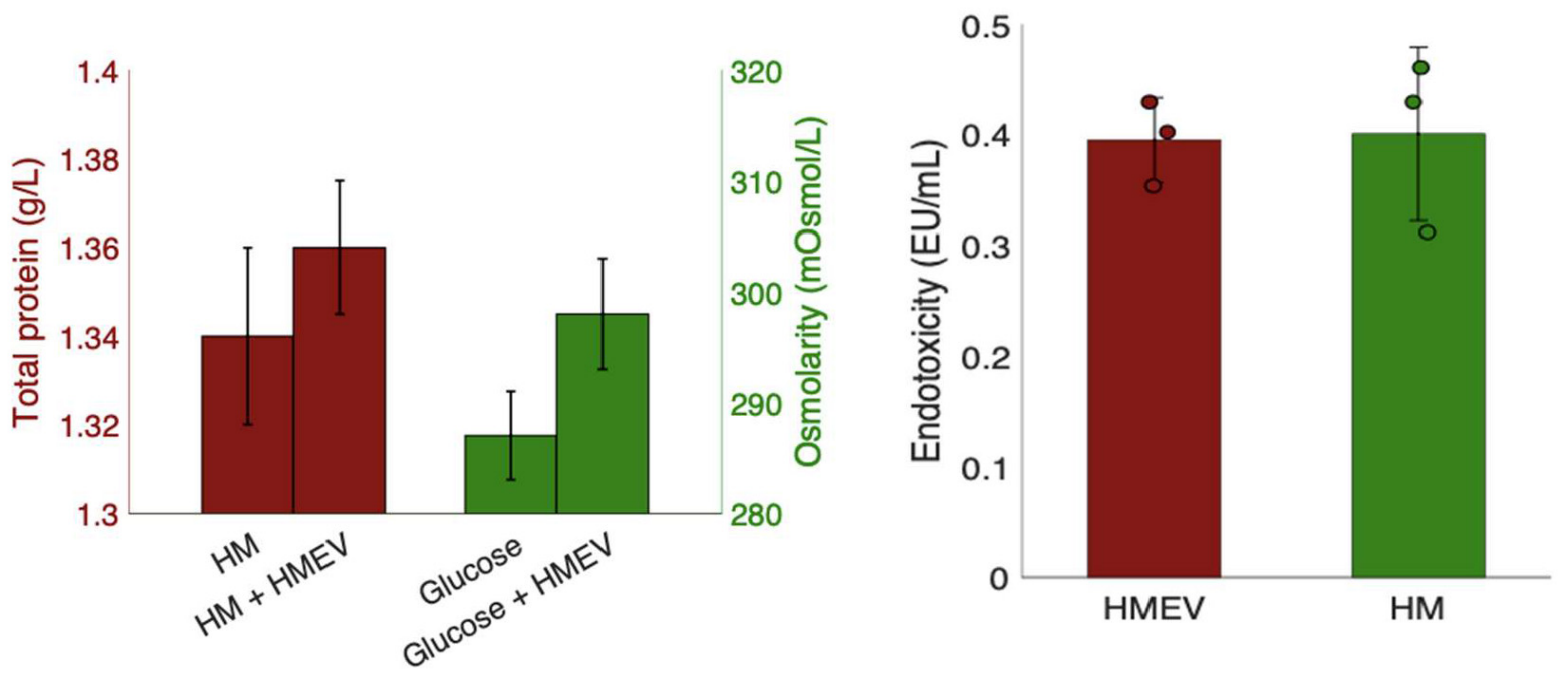
Workflow adaptations for clinical applications. **(Left)**: Triplicate measurements of total protein levels in native and fortified HM (red) and osmolarity of 5% glucose solution with and without HMEVs (green). **(Right)**: Endotoxicity of HMEVs compared to whole HM. Both results were statistically comparable (paired *t*-test, *p*-value > 0.05).

As shown in Figure 6 (left), total protein levels remained comparable between native and EV-fortified HM samples, with no statistically significant differences (Student's *t*-test, *p*-value = 0.05). To ensure that the omission of the filtration step did not affect osmolarity, this parameter was evaluated using three independent HMEV isolates derived from the same pooled HM sample, processed with the final optimized isolation protocol. HMEVs resuspended in 5% glucose solution and analyzed for osmolarity, alongside a control sample consisting of glucose solution alone, maintained osmolarity levels below 380 mOsmol/kg, providing a practical benchmark relevant for future translational evaluation in preterm nutrition contexts (26). Safety was also assessed through microbiological and endotoxicity (Figure 6, right) testing, performed before and after sample handling. All tests yielded negative results.

## Discussion

The main objective of the developed EV isolation workflow was to achieve an HMEV yield comparable to that obtained using the reference method, while improving process efficiency and implementation feasibility. Within the context of this study, which aims to support future evaluation of an HM-derived EV supplementation approach for preterm infants, the co-isolation of naturally occurring HM components was not considered a critical limitation, as these constituents are physiologically compatible and may even contribute beneficial bioactivity.

A significant aspect of the optimization process was the reduction of handling and centrifugation steps. Fewer manipulations decrease vesicle loss and processing time, enhancing workflow practicality for implementation in sterility-oriented settings. The overall results indicate that method 3 provides a favorable compromise between process efficiency and molecular selectivity. In particular, the retention of two 12,000 × *g* centrifugation steps (omitted in method 4) appears to contribute effectively to the removal of non-vesicular material while preserving a streamlined procedure. Characterization by ExoView^®^ confirmed the structural integrity and identity of HMEVs isolated using method 3. The vesicle profiles closely matched those obtained with the reference method, indicating that the optimized workflow preserves vesicle heterogeneity and surface marker diversity. These findings are consistent with previous observations that appropriate ultracentrifugation parameters are crucial for maintaining the native characteristics of milk-derived EVs. The stability assessment demonstrated that HMEVs resuspended in 5% glucose solution remained stable for up to 60 days, confirming the suitability of this medium for both short-term storage and downstream applications. This stability period allows sufficient time for preparation, quality control, and testing in support of future translational studies.

During scale-up experiments, a reduction of approximately 70% in EV recovery was observed when larger rotor volumes were used, highlighting the importance of rotor geometry and loading configuration on sedimentation efficiency. Increasing centrifugation speed to 160,000 × *g* compensated for this loss, improving vesicle recovery by approximately 1.7-fold. This observation emphasizes the necessity of fine-tuning centrifugation parameters during process scaling to maintain optimal yield. Furthermore, omission of the filtration step resulted in a 4.3-fold increase in vesicle concentration and significantly simplified the workflow by reducing handling time and operational complexity. Importantly, ExoView^®^ analysis confirmed that EV integrity and marker composition were not compromised by this modification. Together, these results suggest that filtration can be safely excluded without detriment to EV quality, thus enhancing the feasibility of large-scale production under translationally relevant conditions conditions.

An important consideration for clinical translation is the use of DHM as a limited and valuable clinical resource. In the present study, the workflow was optimized using 378 mL of DHM per batch, resulting in HM-EV–enriched preparations with final particle concentrations in the 10^12^ range and final volumes substantially reduced relative to the starting material (approximately 3 mL per batch). In addition to vesicle-related metrics, the adapted workflow maintained the nutritional integrity and osmolarity of fortified HM within a range commonly considered acceptable in neonatal feeding practice, providing a practical benchmark relevant for future translational evaluation. Osmolarity values consistently below 380 mOsmol/kg comply with neonatal feeding guidelines, minimizing the risk of gastrointestinal complications associated with hyperosmolar feeds. Microbiological screening and endotoxin testing further confirmed the safety of the product, demonstrating that the workflow is compatible with sterility-oriented handling and testing environments.

Compared with previously reported EV isolation strategies from biological fluids, the present method represents a substantial improvement in balancing vesicle yield, purity, and implementation feasibility for larger-batch processing. The streamlined protocol reduces both time and equipment demand, facilitating its translation from research laboratories to more routine processing environments. The demonstrated stability, safety, and reproducibility of HMEV-enriched preparations underline their potential for further evaluation as a novel component of nutritional support for preterm infants. Collectively, these findings support method 3 as a robust, scalable, and translationally oriented approach for HMEV isolation. Beyond nutritional supplementation, the optimized workflow may also serve as a foundation for future development of milk-derived EV-based therapeutic formulations, expanding the translational potential of HM bioactive formulations.

## Conclusions

An optimized workflow for HMEV isolation and fortification was established providing methodological groundwork for future translational studies in neonatal nutrition. The method preserves HMEV structural integrity, maintains nutritional quality and osmolarity within neonatal safety limits, and ensures microbiological safety suitable for future *in vivo* studies. This method will be applied in an upcoming interventional pilot trial to evaluate the tolerance and safety of repeated HMEV supplementation, co-administered with MOM for two weeks in preterm newborns. Vesicles will be isolated from a volume of DHM corresponding to the average daily intake of HM in preterm infants on full enteral nutrition. This represents a pioneering step toward translating the preventive properties of HMEVs into tangible health outcomes for highly vulnerable patients.

## Data Availability

The raw data supporting the conclusions of this article will be made available by the authors, without undue reservation.
