# Supplementary material for: Isolation and characterization of extracellular vesicles from human milk for potential use as a dietary supplement in clinical research with preterm infants
Source: Front Nutr. 2026 Mar 20;13:1764928. doi: 10.3389/fnut.2026.1764928 (PMC13046510; doi:10.3389/fnut.2026.1764928)
Supplement: Supplementary file 1 [file Data_Sheet_1.pdf]

Supplementary material to: Isolation and characterization of Extracellular Vesicles from Human Milk for potential use as a dietary supplement in clinical research with preterm infants

Jose Luis Moreno-Casillas[1,2,¥], Laura Ripoll-Seguer[1,2, ¥], Emmanuel Rumba-Matano[1,2], Anna Parra-Llorca[3,4], María Cernada[3,4], María Jesús Vaya[5], Eduardo López Briz[6], Ana María Padilla- López[6], Ana Gil Brusola[7], Bernhard Lendl[8], Guillermo Quintás[9], María Gormaz[3,4], Julia Kuligowski[2,3]

[1]*Neonatal Research Group, Health Research Institute Hospital La Fe (IIS La Fe), Avda Fernando Abril Martorell 106, 46026 Valencia, Spain*

[2]*Servicio de Análisis de Vesículas Extracelulares (SAVE), Health Research Institute Hospital La Fe (IIS La Fe), Avda Fernando Abril Martorell 106, 46026, Valencia, Spain*

[3]*Spanish Network in Maternal, Neonatal, Child and Developmental Health Research (RICORS- SAMID) (RD24/0013/0014), Neonatal Research Group, Health Research Institute La Fe, Avda Fernando Abril Martorell 106, 46026 Valencia, Spain*

[4]*Division of Neonatology, University & Polytechnic Hospital La Fe (HULAFE), Avda Fernando Abril Martorell 106, 46026 Valencia, Spain*

[5]Blood Transfusion Center from the Valencian Community, Avda del Cid, 65-acc, 46014, Valencia, Spain

[6]Hospital Pharmacy Department, University and Polytechnic Hospital La Fe, Avenida Fernando Abril Martorell, 106 Torre B, 46026 Valencia, Spain

[7]Department of Microbiology, University and Polytechnic Hospital La Fe, *), Avda Fernando Abril Martorell 106, 46026 Valencia, Spain*

[8]*Institute of Chemical Technologies and Analytics, Technische Universität Wien, Getreidemarkt 9/164, A 1060, Vienna, Austria*

[9]*Health and Biomedicine, Leitat Technological Center, Carrer de la Innovació, 2, 08225 Terrassa, Spain*

[¥]both authors contributed equally

*Corresponding author: Julia Kuligowski PhD; Avda Fernando Abril Martorell 106, 46026 Valencia, Spain; Phone: +34/961246661; e-mail: julia.kuligowski@uv.es | julia_kuligowski@iislafe.es

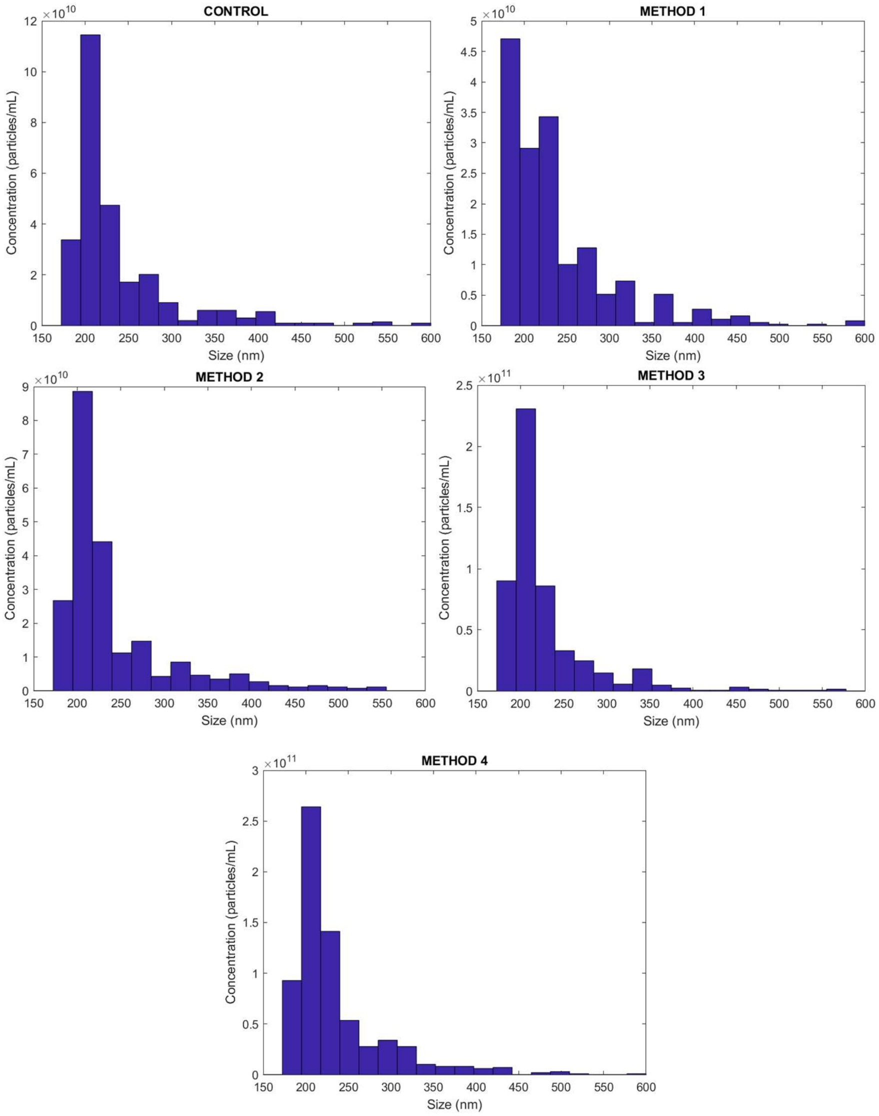

Figure S1. Size distribution and particle concentration histograms for control, and methods 1 to 4.

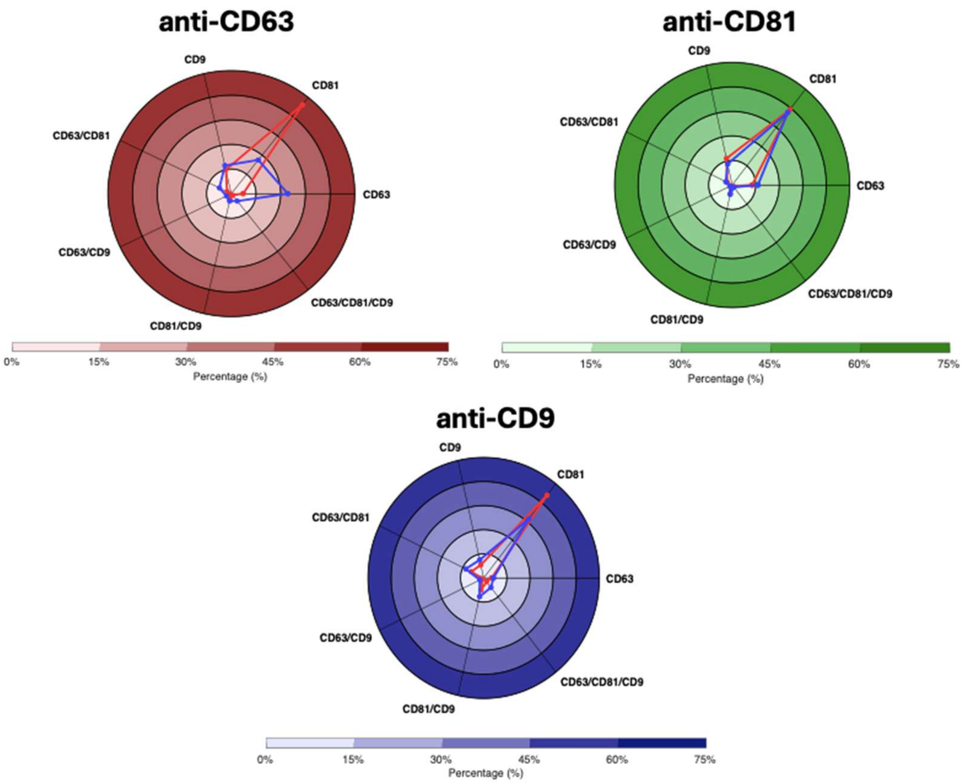

Figure S2. Relative abundance of tetraspanin-positive HMEVs (CD63, CD81, CD9) captured and labeled employing the ExoView® platform. Note: Control – blue and method 3 – red.

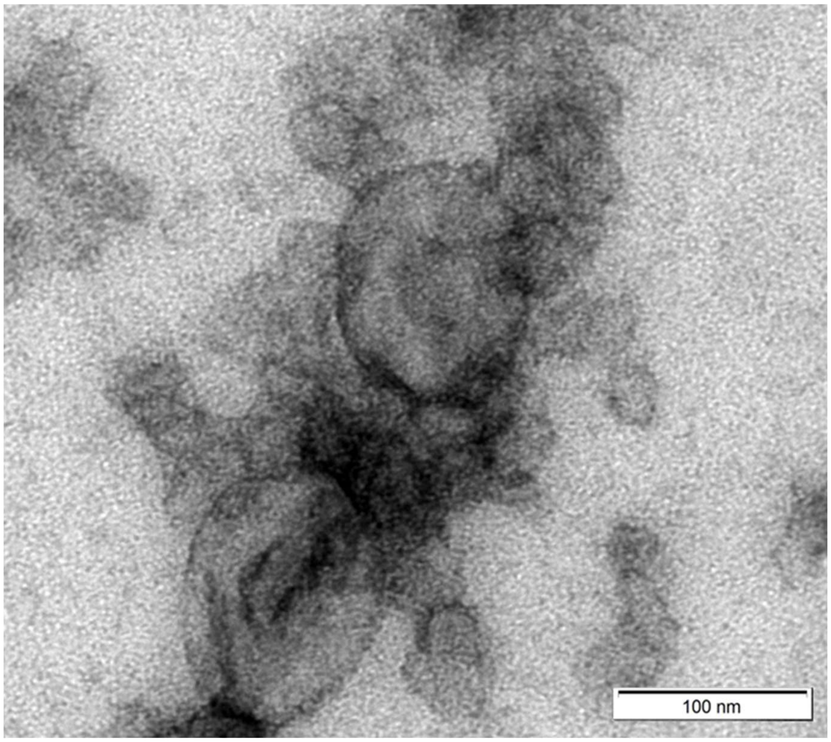

Figure S3. TEM image of HMEVs isolated using method 3.

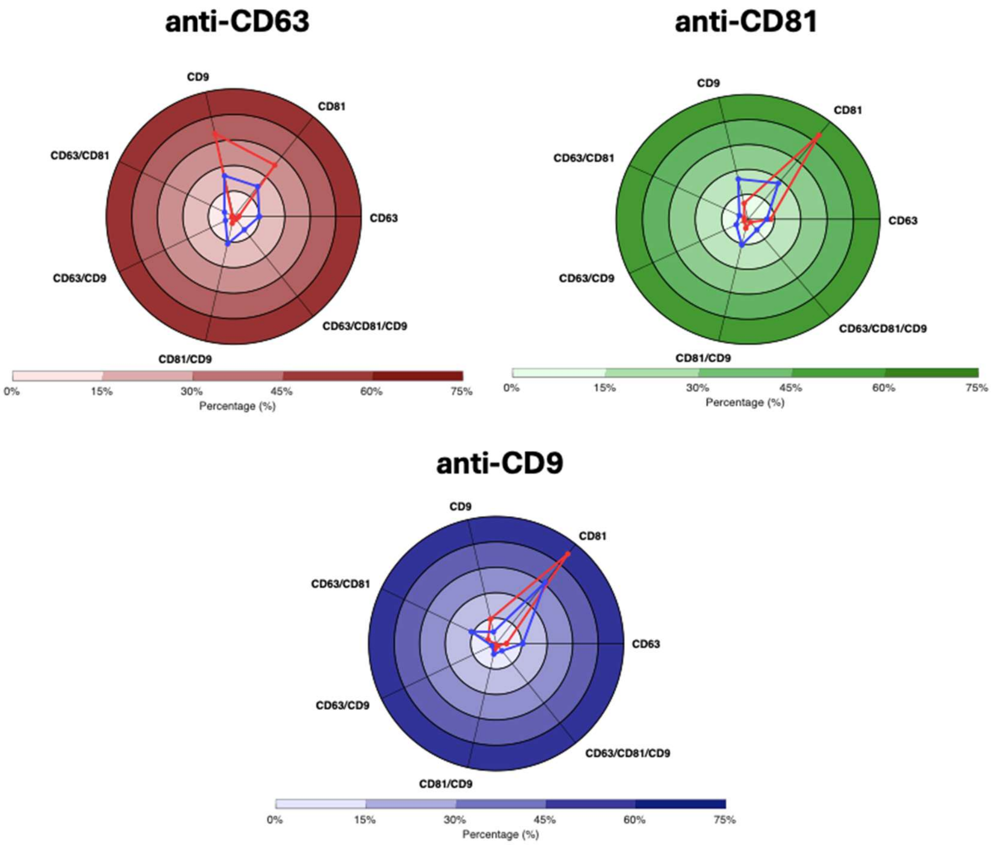

Figure S4. Relative abundance of tetraspanin-positive HMEV (CD63, CD81, CD9) captured and labeled employing the ExoView® platform. Note: Filtered – red and Unfiltered – blue.

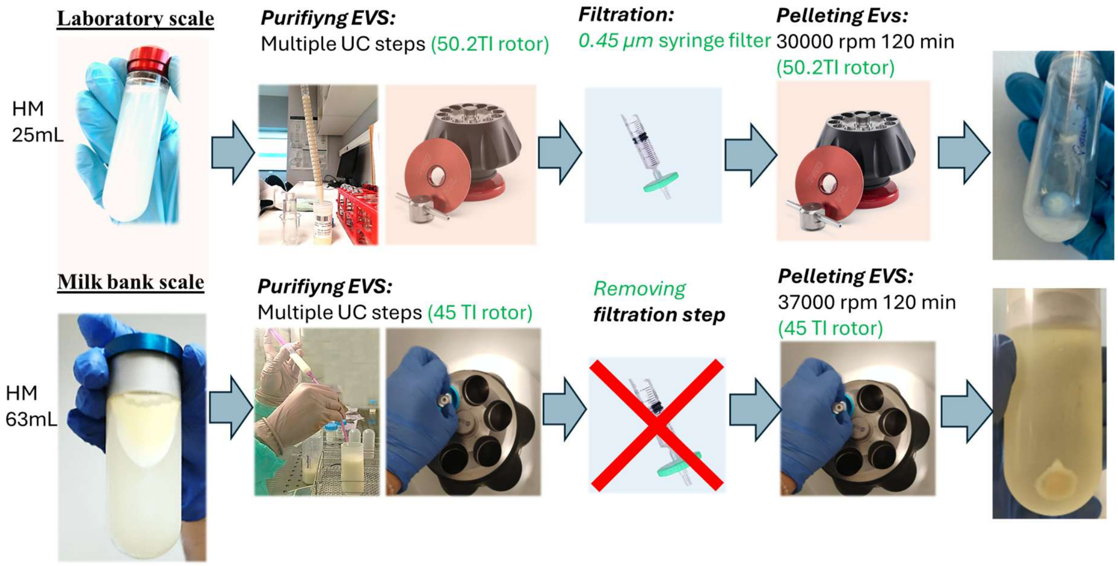

Figure S5. Schematic representation of the laboratory-scale (top) and milk bank-scale (bottom) HMEV isolation procedures. Key differences include the use of different rotors (50.2 Ti vs 45 Ti) and tubes (26.3 mL vs 94 mL), the removal of the filtration step, and an increased centrifugation speed to 160000 x g in the milk bank-scale process.
